# Supplementary material for: Implications of kappa-casein evolutionary diversity for the self-assembly and aggregation of casein micelles
Source: R Soc Open Sci. 2019 Oct 16;6(10):190939. doi: 10.1098/rsos.190939 (PMC6837221; doi:10.1098/rsos.190939)
Supplement: Supplementary material [file rsos190939supp1.pdf]

Implications of kappa-casein evolutionary diversity for the self-assembly and aggregation of casein micelles

Supplementary Material – Royal Society Open Science, 2019

Jean Manguy[1, 2, 3] and Denis C. Shields[1, 2, 3, *]

[1]UCD Conway Institute, University College Dublin, Belfield, Dublin 4, Ireland
[2]School of Medicine, University College Dublin, Belfield, Dublin 4, Ireland
[3]Food for Health Ireland, University College Dublin, Belfield, Dublin 4, Ireland
[*]Corresponding author: denis.shields@ucd.ie

List of Figures

S1 Species tree and representation of the kappa-casein protein sequences alignment. 2
S2 Time-period specific variation in the rate of evolution of kappa-casein 3
S3 Clade specific indels in kappa-casein 3
S4 Amino acid frequencies scatter plot between PKC and GMP 4
S5 Distribution of phophorylated serines and O-glycosylated serines and threonines in kappa-casein sequences. 5
S6 Distribution of cysteines in kappa-casein sequences. 6

List of Tables

S1 Kappa-casein GenInfo Identifiers 7
S2 Protein tandem repeats found in kappa-casein sequences. 10
S3 Prediction of O-glycosylation in kappa-casein 11

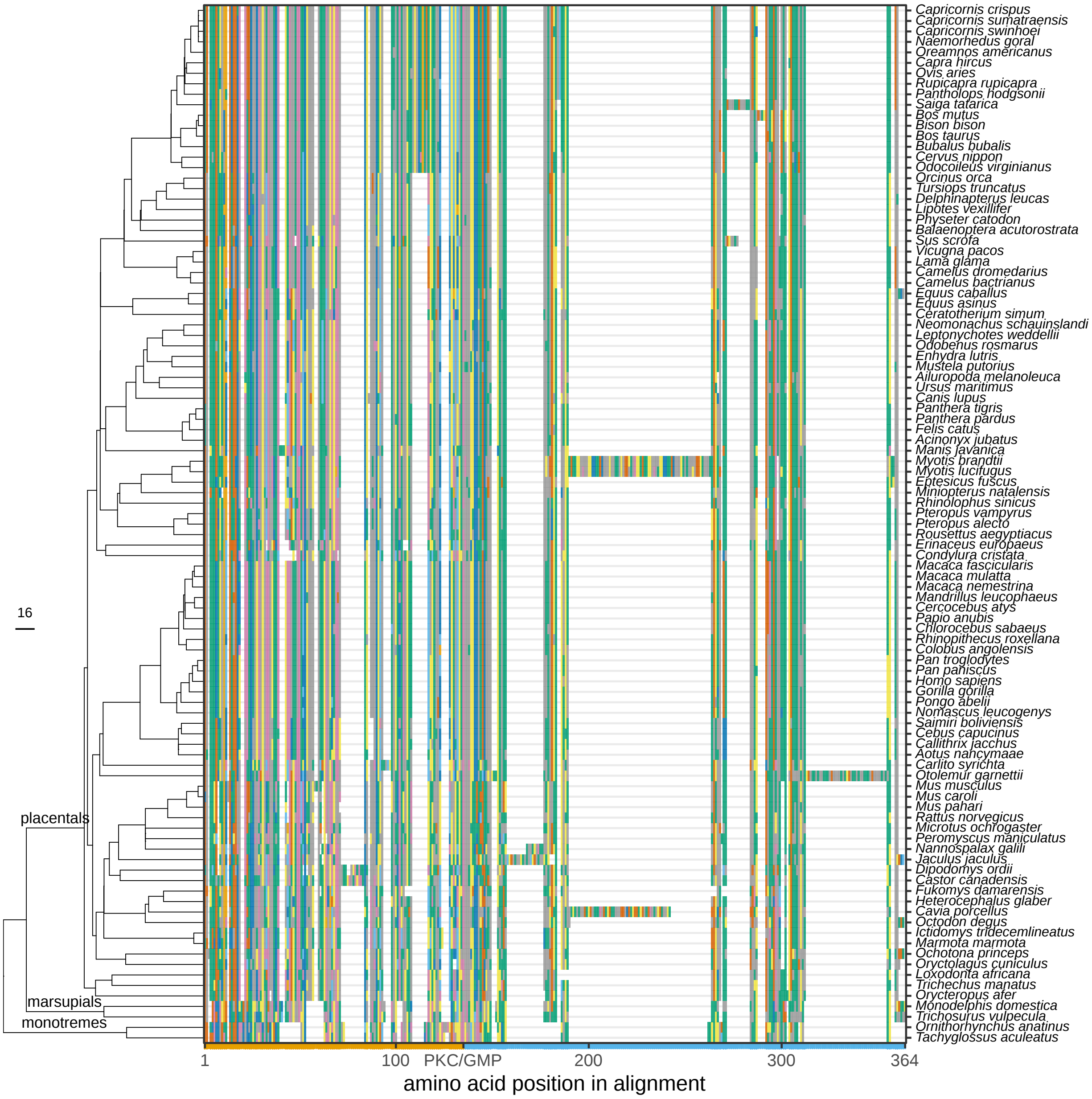

Figure S1: Species tree and representation of the kappa-casein protein sequences alignment. The branch length of the species tree are given in million of years. Grey: hydrophobic (ILVAM). Green: polar (STNQ). Pink: aromatic (YFW). Orange: cysteine. Yellow: proline. Blue: positively charged (HRK). Red: negatively charged (DE). Below the plot, the orange line represents PKC, and the blue line represents GMP.

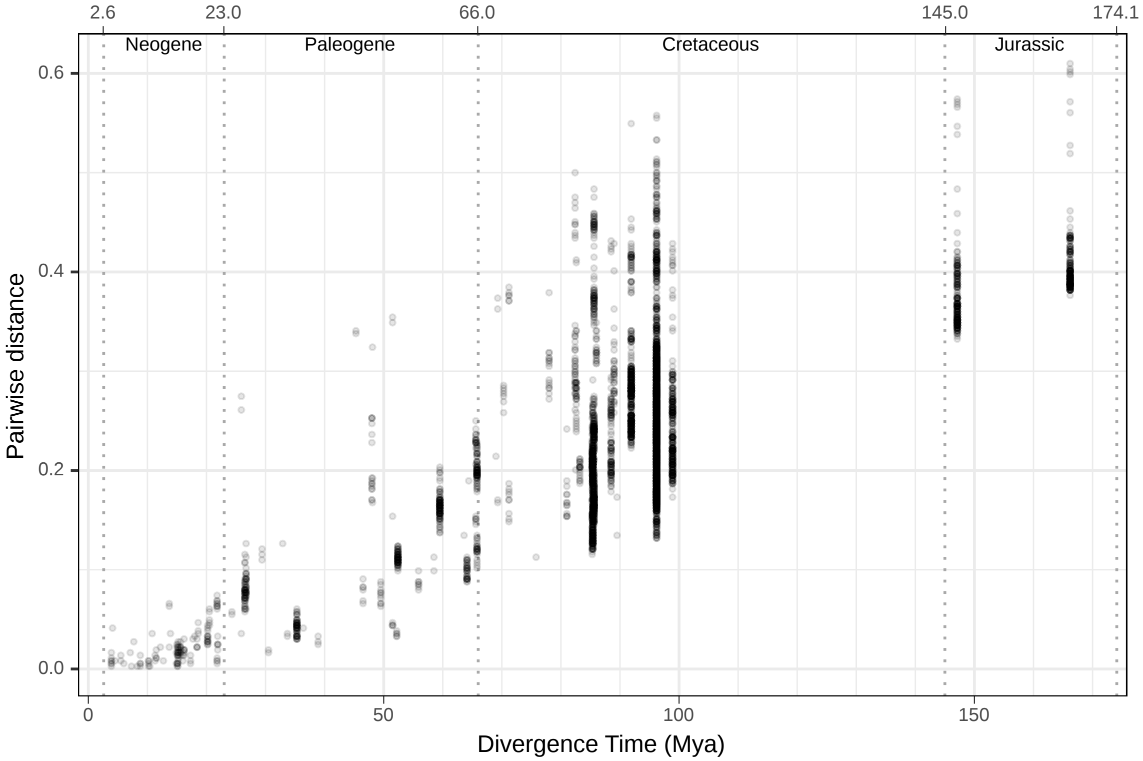

Figure S2: Time-period specific variation in the rate of evolution of kappa-casein. Scatter plot of the pairwise distance between mature kappa-casein sequences (without correction for multiple testing) and the divergence time.

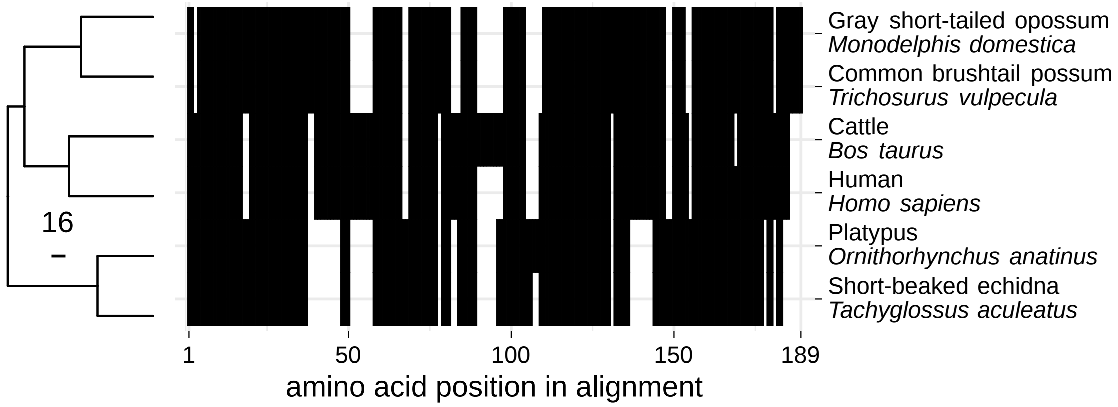

Figure S3: Clade specific indels in some representative sequences of kappa-casein The black bars represent any amino acid in an aligned sequence of mature kappa-casein. While the absence of bar represents a gap. Cow (*Bos taurus*) and human (*Homo sapiens*) sequences were arbitrary selected to represent the Eutheria infraclass; the platypus (*Ornithorhynchus anatinus*) and the short-beaked echidna (*Tachyglossus aculeatus*) sequences represent the Prototheria subclass; the common brushtail possum (*Trichosurus vulpecula*) and the grey short-tailed opossum sequences (*Monodelphis domestica*) represent the Metatheria infraclass.

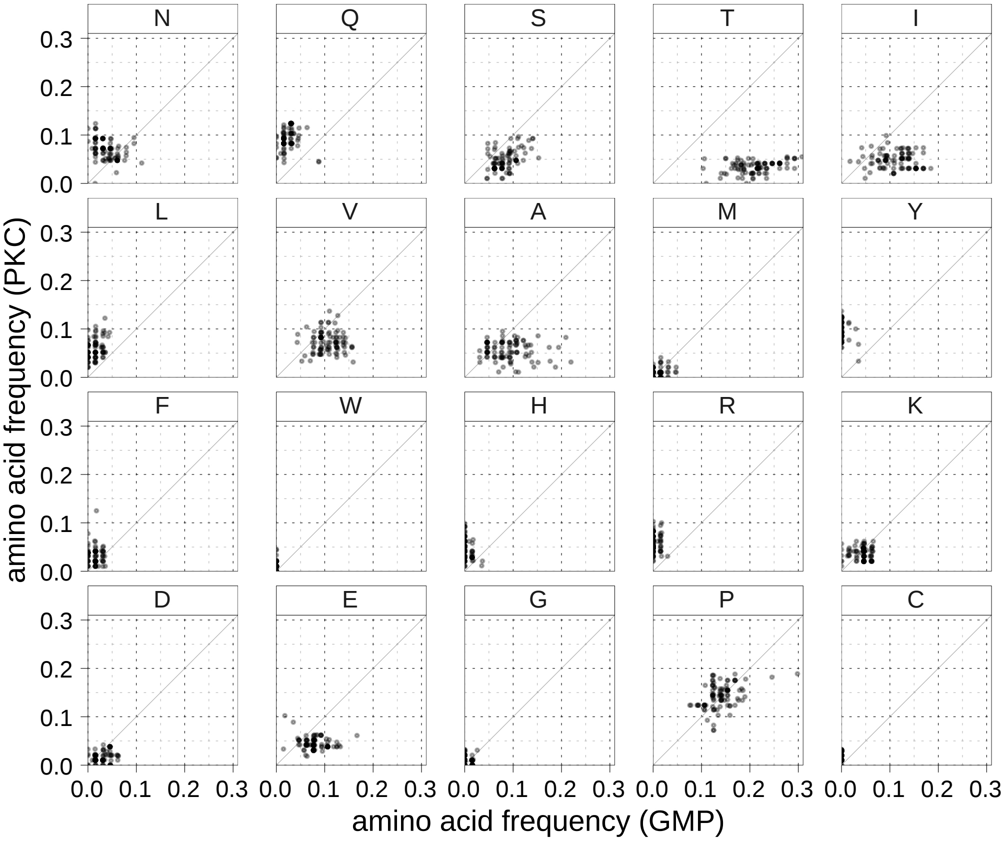

Figure S4: Amino acid frequencies scatter plot between PKC and GMP. The frequency of each amino acid for each para-kappa-casein (PKC) and glyco-macro-peptide (GMP) sequence is represented by a point.

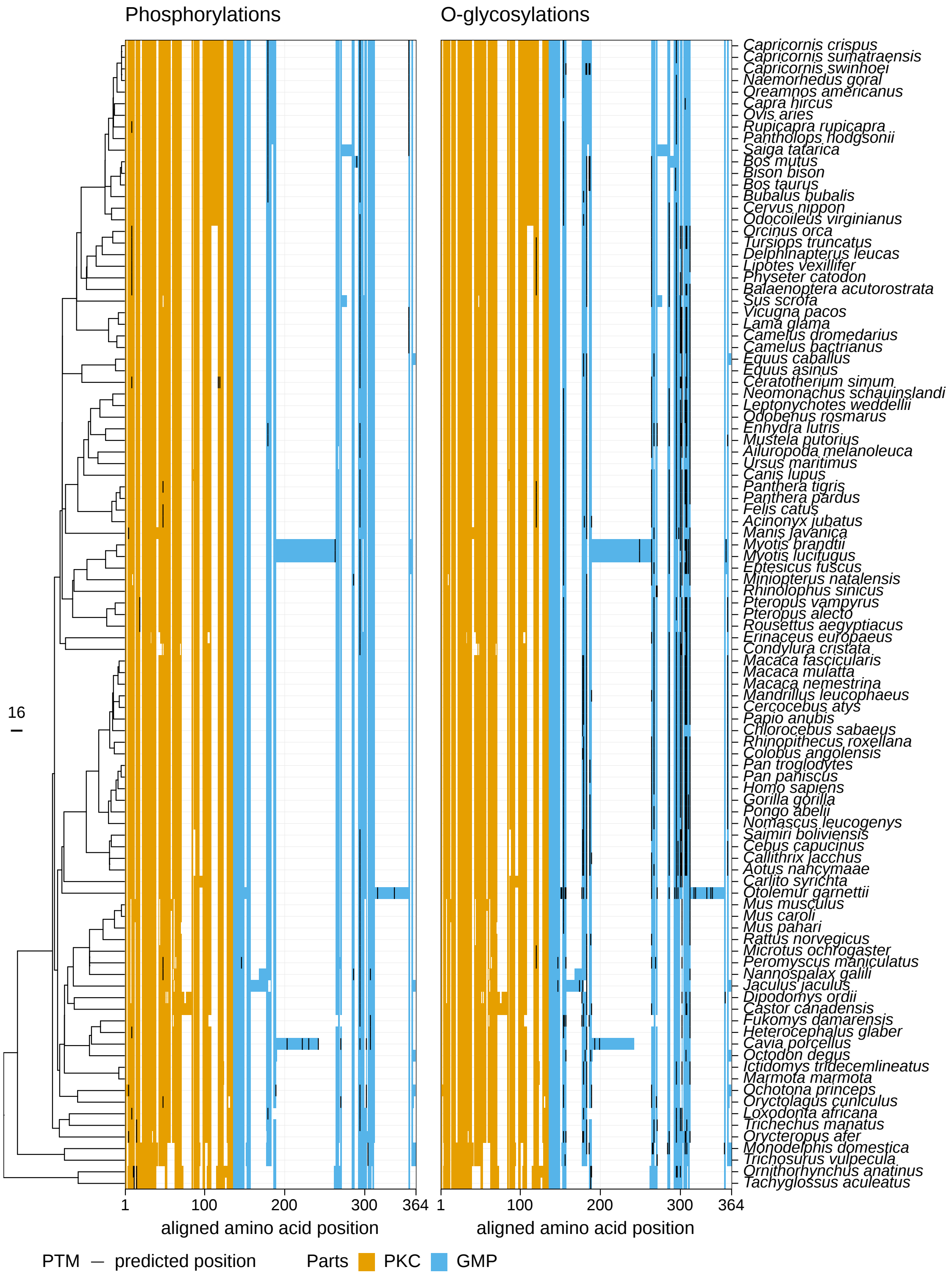

Figure S5: Distribution of phophorylated serines and O-glycosylated serines and threonines in kappa-casein sequences. The vertical line represents the predicted PTM. Coloured bars represent the part of kappa-casein sequences: PKC in yellow and GMP in blue. On the side, a pruned mammalian tree is shown [1]. Phosphorylations predictions were performed using a regular expression of the fam20c cannonical motif [2]. Glycosylation predictions were performed with GlycoMine (cut-off = 0.4) [3].

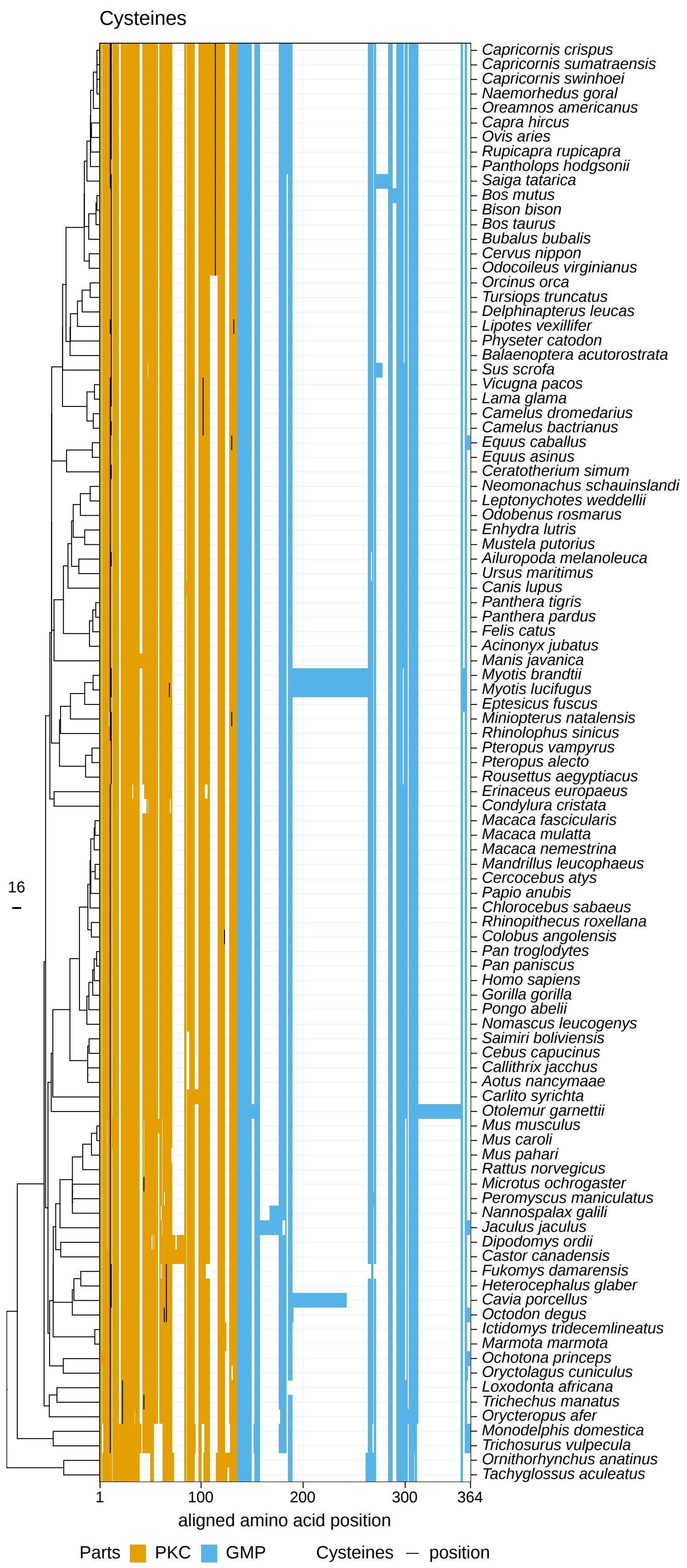

Figure S6: Distribution of cysteines in kappa-casein sequences. The vertical black lines represent cysteines. Coloured bars represent the part of kappa-casein sequences: PKC in yellow and GMP in blue. On the side, a pruned mammalian tree is shown [1].

Table S1: Kappa-casein GenInfo Identifiers.

species	NCBI Taxon ID	NCBI GI Identifier
Tachyglossus aculeatus	9261	255661244
Ornithorhynchus anatinus	9258	255661248
Trichosurus vulpecula	9337	4559294
Monodelphis domestica	13616	126330606
Orycteropus afer	1230840	634853626
Trichechus manatus	127582	[a]
Loxodonta africana	9785	731494712
Oryctolagus cuniculus	9986	1607
Ochotona princeps	9978	504173483
Marmota marmota	9994	984137438
Ictidomys tridecemlineatus [b]	43179	532095382
Octodon degus	10160	507713885
Cavia porcellus	10141	115670
Heterocephalus glaber	10181	351699116
Fukomys damarensis [c]	885580	1104935430
Castor canadensis	51338	1147391913
Dipodomys ordii	10020	852759962
Jaculus jaculus	51337	507563375
Nannospalax galili [d]	1026970	674032426
Peromyscus maniculatus	230844	1008789005
Microtus ochrogaster	79684	532033496
Rattus norvegicus	10116	13928762
Mus pahari	10093	1195508802
Mus caroli	10089	1195722227
Mus musculus	10090	75677412
Otolemur garnettii	30611	395857266
Carlito syrichta [e]	1868482	640822240
Aotus nancymaae	37293	817316329
Callithrix jacchus	9483	1060981464
Cebus capucinus	1737458	1044411177
Saimiri boliviensis	39432	403280959
Nomascus leucogenys	61853	332233119
Pongo abelii	9601	297673660
Gorilla gorilla	9595	426344545
Homo sapiens	9606	29676
Pan paniscus	9597	397475232
Pan troglodytes	9598	114594392
Colobus angolensis	336983	795340768
Rhinopithecus roxellana	61622	724803799
Chlorocebus sabaeus	60711	635042818
Papio anubis	9555	402869633
Cercocebus atys	9531	795383260
Mandrillus leucophaeus	9568	795308736
Macaca nemestrina	9545	795623958
Macaca mulatta	9544	109074586
Macaca fascicularis	9541	355749359
Condylura cristata	143302	507942890
Erinaceus europaeus	9365	617605017
Rousettus aegyptiacus	9407	1012258563

(continued …)

Table S1: Kappa-casein GenInfo Identifiers. *(continued)*

species	NCBI Taxon ID	NCBI GI Identifier
Pteropus alecto	9402	586524827
Pteropus vampyrus	132908	759121339
Rhinolophus sinicus	89399	1124008234
Miniopterus natalensis	291302	1016674193
Eptesicus fuscus	29078	641730880
Myotis lucifugus	59463	940768390
Myotis brandtii	109478	946799019
Manis javanica	9974	1048452318
Acinonyx jubatus	32536	961710667
Felis catus	9685	410957476
Panthera pardus	9691	1111079331
Panthera tigris	74533	591321662
Canis lupus	9615	545521723
Ursus maritimus	29073	671001068
Ailuropoda melanoleuca	9646	1126262988
Mustela putorius	9669	859857021
Enhydra lutris	391180	1244101367
Odobenus rosmarus	9708	472346437
Leptonychotes weddellii	9713	585154038
Neomonachus schauinslandi [f]	29088	1212216747
Ceratotherium simum	73337	955478944
Equus asinus	9793	958718360
Equus caballus	9796	19031197
Camelus bactrianus	9837	429534184
Camelus dromedarius	9838	1742992
Lama glama	9844	787034497
Vicugna pacos [g]	30538	560980638
Sus scrofa	9823	55742766
Balaenoptera acutorostrata	310752	594692663
Physeter catodon	9755	593761147
Lipotes vexillifer	118797	602729614
Delphinapterus leucas	9749	1246240428
Tursiops truncatus	9739	470658938
Orcinus orca	9733	466001209
Odocoileus virginianus	9880	1187550739
Cervus nippon	9863	295705
Bubalus bubalis	89462	295701
Bos taurus	9913	1228078
Bison bison	43346	742114810
Bos mutus [h]	72004	440904989
Saiga tatarica	34875	1033241
Pantholops hodgsonii	59538	556777482
Rupicapra rupicapra	34869	1033238
Ovis aries	9940	57164381
Capra hircus	9925	978
Oreamnos americanus	34873	1033235
Naemorhedus goral	34871	1033232
Capricornis swinhoei	34866	1033201
Capricornis sumatraensis	34865	1033203

(continued ...)

Table S1: Kappa-casein GenInfo Identifiers. *(continued)*

species	NCBI Taxon ID	NCBI GI Identifier
Capricornis crispus	9966	295703

[a] First exon was recovered from a BLAST with the elephant sequence
[b] Was “Spermophilus tridecemlineatus” in Fritz, Bininda-Emonds and Purvis [1]
[c] Was “Cryptomys damarensis” in Fritz, Bininda-Emonds and Purvis [1]
[d] Used instead of “Spalax ehrenbergi” in Fritz, Bininda-Emonds and Purvis [1]
[e] Was “Tarsius syrichta” in Fritz, Bininda-Emonds and Purvis [1]”
[f] Was “Monachus schauinslandi" in Fritz, Bininda-Emonds and Purvis [1]”
[g] Was “Vicugna vicugna” in Fritz, Bininda-Emonds and Purvis [1]
[h] Was “Bos grunniens” in Fritz, Bininda-Emonds and Purvis [1]

Table S2: Protein tandem repeats found in kappa-casein sequences. Positions are given for the extremities of each tandem repeat unit in the mature sequence.

	position		
species	N	C	sequence
Cavia porcellus	133	145	SAGDTPEVSSQFI
Cavia porcellus	146	171	DTPDTSVLAEEARESPEDTPEISEFI
Cavia porcellus	172	198	NAPDTAVPSEEPRESAEDTPEISSEFI
Castor canadensis	51	62	INNPYMPYPYYV
Castor canadensis	63	74	ISNPYMSYPYYS
Dipodomys ordii *	48	62	INSPYMPFPYYA
Dipodomys ordii *	63	69	VNNLPYTYST
Jaculus jaculus	101	112	NADPNASAIPSA
Jaculus jaculus	113	124	NAHPDASAIPSA
Jaculus jaculus	125	136	NAHPDASAIPSP
Otolemur garnettii	112	114	PTT
Otolemur garnettii	115	117	PTI
Otolemur garnettii	141	161	PETSSVSAVTNTLEAAAVTVT
Otolemur garnettii	162	182	PEASSVSAITNTLEAAAVTVT
Otolemur garnettii	183	203	PEASSVSAVTNTLEAAAVTVT
Myotis lucifugus	95	131	PSLFAIPPKKNQDKAVIPTANTVPADEPTLIPPSEST
Myotis lucifugus	132	168	PPLFAVPPKKNQDKAVIPIVNTVPADEATLFPPSEST
Myotis lucifugus	169	205	PPLFATPPKKNQDKAVIPTINIIPADEPTVILSSEPT
Myotis brandtii	95	131	PSLFAIPPKKNQDKAVIPTANTVPADEPTLIPPSEST
Myotis brandtii	132	168	PPLIAIPPKKNQDKAVIPIVNTVPADEPTLFPPSEST
Myotis brandtii	169	205	PPLIAIPPKKNQDKAVIPTINIIAADEPTVILSSEPT
Manis javanica	33	35	NSL
Manis javanica	36	38	NSS
Sus scrofa	128	133	EPIVNA
Sus scrofa	134	139	EPIVNA
Bos mutus	147	150	EASP
Bos mutus	151	154	EASP
Saiga tatarica	137	142	EAIVNT
Saiga tatarica	143	148	EAIVNT
Saiga tatarica	149	154	EAIVNT

* likely highly degenerate repeat

Table S3: Prediction of O-glycosylation in kappa-casein with GlycoMine [3]; GlycoPred [4]; O-GlcNAcPRED-II [5]; and NetOGlyc4.0 [6].

method	sensivity	specificity	precision	accuracy	MCC
GlycoMine	0.75	0.95	0.86	0.89	0.72
GlycoPred	0.94	0.32	0.38	0.51	0.28
O-GlcNAcPRED-II	0.56	0.62	0.39	0.60	0.17
NetOClyc4.0	0.56	0.54	0.35	0.55	0.09

References

[1] Susanne A. Fritz, Olaf R. P. Bininda-Emonds and Andy Purvis. 'Geographical Variation in Predictors of Mammalian Extinction Risk: Big Is Bad, but Only in the Tropics'. en. In: *Ecology Letters* 12.6 (June 2009), pp. 538–549. ISSN: 1461-0248. DOI: 10.1111/j.1461-0248.2009.01307.x.

[2] Vincent S. Tagliabracci et al. 'Secreted Kinase Phosphorylates Extracellular Proteins That Regulate Biomineralization'. en. In: *Science* 336.6085 (June 2012), pp. 1150–1153. ISSN: 0036-8075, 1095-9203. DOI: 10.1126/science.1217817.

[3] Fuyi Li et al. 'GlycoMine: A Machine Learning-Based Approach for Predicting N-, C- and O-Linked Glycosylation in the Human Proteome'. en. In: *Bioinformatics* 31.9 (May 2015), pp. 1411–1419. ISSN: 1460-2059, 1367-4803. DOI: 10.1093/bioinformatics/btu852.

[4] Stephen E. Hamby and Jonathan D. Hirst. 'Prediction of Glycosylation Sites Using Random Forests'. In: *BMC Bioinformatics* 9.1 (Nov. 2008), p. 500. ISSN: 1471-2105. DOI: 10.1186/1471-2105-9-500.

[5] Cangzhi Jia et al. 'O-GlcNAcPRED-II: An Integrated Classification Algorithm for Identifying O-GlcNAcylation Sites Based on Fuzzy Undersampling and a K-Means PCA Oversampling Technique'. en. In: *Bioinformatics* 34.12 (June 2018), pp. 2029–2036. ISSN: 1367-4803. DOI: 10.1093/bioinformatics/bty039.

[6] Catharina Steentoft et al. 'Precision Mapping of the Human O-GalNAc Glycoproteome through SimpleCell Technology'. eng. In: *The EMBO journal* 32.10 (May 2013), pp. 1478–1488. ISSN: 1460-2075. DOI: 10.1038/emboj.2013.79.
